# VKECE-3D: Energy-Efficient Coverage Enhancement in Three-Dimensional Heterogeneous Wireless Sensor Networks Based on 3D-Voronoi and K-Means Algorithm

**DOI:** 10.3390/s23020573

**Published:** 2023-01-04

**Authors:** Pingzhang Gou, Baoyong Guo, Miao Guo, Shun Mao

**Affiliations:** College of Computer Science and Engineering, Northwest Normal University, Lanzhou 730070, China

**Keywords:** heterogeneous wireless sensor networks, highly destructive polynomial mutation strategy, 3D-Voronoi partitioning, K-means clustering algorithm, polling working mechanism

## Abstract

During these years, the 3D node coverage of heterogeneous wireless sensor networks that are closer to the actual application environment has become a strong focus of research. However, the direct application of traditional two-dimensional planar coverage methods to three-dimensional space suffers from high application complexity, a low coverage rate, and a short life cycle. Most methods ignore the network life cycle when considering coverage. The network coverage and life cycle determine the quality of service (QoS) in heterogeneous wireless sensor networks. Thus, energy-efficient coverage enhancement is a significantly pivotal and challenging task. To solve the above task, an energy-efficient coverage enhancement method, VKECE-3D, based on 3D-Voronoi partitioning and the K-means algorithm is proposed. The quantity of active nodes is kept to a minimum while guaranteeing coverage. Firstly, based on node deployment at random, the nodes are deployed twice using a highly destructive polynomial mutation strategy to improve the uniformity of the nodes. Secondly, the optimal perceptual radius is calculated using the K-means algorithm and 3D-Voronoi partitioning to enhance the network coverage quality. Finally, a multi-hop communication and polling working mechanism are proposed to lower the nodes’ energy consumption and lengthen the network’s lifetime. Its simulation findings demonstrate that compared to other energy-efficient coverage enhancement solutions, VKECE-3D improves network coverage and greatly lengthens the network’s lifetime.

## 1. Introduction

With the evolution of communication technologies, information-centric wireless communication and edge computing are becoming increasingly popular in 5G and the IoT. Wireless sensor networks (WSNs) are a common example of them. It is centered around a distributed base station, where wireless sensor nodes are the devices of the IoT, and each node uses wireless data communication to exchange information with one another. WSNs are multi-hop self-organizing networks made up of sensor nodes, tiny devices, and other devices that monitor occurrences in a target area and communicate the gathered data to a data center for processing [1]. In practical wireless sensor network application scenarios, heterogeneous wireless sensor networks (HWSNs) are often formed due to differences in the sensing, communication, and processing speeds of the nodes. As communication and software technologies advance, HWSNs have become a major focus in ecological environment detection [2], agricultural research [3], military research [4], and disaster relief [5]. The small size, low energy consumption, and portability of the nodes determine their rich and diverse usage scenarios. However, in the process of actual deployment, due to their uneven random deployment, node communication failure, and malicious network attacks, some nodes can fail and can generate coverage holes, which in turn reduce the monitoring quality and the network life cycle [6]. In addition, large-scale high-density node deployment may cause coverage overlap, resulting in data collection transmission redundancy and channel interference [7], thus wasting limited energy. Therefore, energy-efficient coverage enhancement is a critical and challenging issue for HWSNs. It is important to extend the network life cycle by using suitable coverage enhancement algorithms and node scheduling strategies to make some nodes dormant with guaranteed coverage.

The study of the 2D flat coverage enhancement problem has been carried out earlier and has achieved rich achievements. However, in practical coverage tasks, nodes are commonly deployed in complex three-dimensional environments. To meet current practical needs, the study of the three-dimensional coverage enhancement problem needs to be further deepened urgently [8]. Most existing methods use swarm-intelligence algorithms to enhance network coverage quality but often ignore the network’s life cycle. For the problem of the high aggregation and poor uniformity of random deployment nodes, a suitable secondary deployment strategy can be used to solve the problem [9]. According to the idea of clustering and computational geometry, the target area is partitioned into different clusters, and a method that calculates the optimal sensing radius of different structures is used to form HWSNs clusters in which nodes move to the center–of–mass position, which can reduce the network average movement distance. For a redundant node dormancy mechanism, a common approach is to use more than one sensor node working together to monitor the common goal, and then to establish a rotational dormancy and wake-up mechanism between sensor nodes to enhance the node energy efficiency, resulting in lower network energy consumption [10]. Zhao et al. [11] used the Vampire Bat Optimizer (VBO) to redeploy the nodes and effectively enhance the network coverage. Feng et al. [12] combined a clustering algorithm with the Artificial Fish-Swarm Algorithm (AFSA) to improve the network monitoring quality while ensuring maximum network coverage and minimizing energy consumption. Wang et al. [13] improved the coverage and extended the network life cycle by switching redundant nodes to static or sleep mode based on a grid partition strategy and calculating the mobile destinations of nodes by taking advantage of the Particle Swarm Optimization (PSO) algorithm. Thus, this research comes up with an energy-efficient coverage enhancement method for three-dimensional environments combining the above strategies.

**Figure 1:**
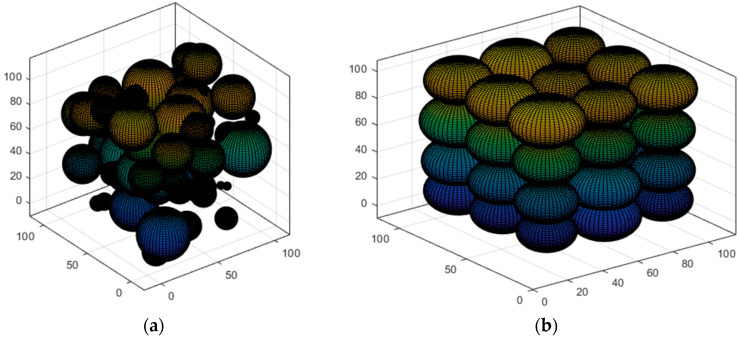
3D-coverage enhancement scene: (**a**) Random deployment; (**b**) After coverage enhancement.

We use cubic space to simulate the 3D deployment scenario in the field. According to Figure 1, the network suffers from a high degree of aggregation after random deployment. The coverage is optimized using a suitable scheme. To lower the deployed quantity of nodes and minimize the energy consumption of a single node, the sensing radius of the nodes can be made to adjust adaptively because of the actual demands. In turn, the network’s QoS can be improved. Subsequent research is carried out based on this scenario.

Coverage enhancement is the classical research of HWSNs. In this research, we propose solutions for 3D energy-efficient coverage enhancement scenarios. To lower the energy consumption of deployed nodes, 3D-Voronoi partitioning is combined with the K-means algorithm, and multi-hop communication and polling working mechanisms are introduced. The mobile nodes are grouped around the cell’s center of mass, and the distance between the center of mass and the cell border is chosen as the ideal sensing radius of the nodes, lowering the quantity of active nodes, and therefore extending the network’s life cycle while ensuring coverage quality. The following are the primary contributions to this research.

- To improve the network homogeneity, a secondary deployment of nodes using a highly destructive polynomial mutation strategy is proposed based on the idea of mutation characteristics;
- After the elbow method determines the quantity of clusters k, the nodes after secondary deployment are divided into several clusters using K-means, and the optimal sensing radius of the computational unit is calculated using 3D-Voronoi partition to lower the quantity of active nodes and improve the network’s QoS;
- Network data communication is divided into single-hop communication and multi-hop communication. The polling working mechanism is established according to the distance of the centroid from near to far. All nodes outside the unit centroid are dormant to lower the nodes’ energy consumption and lengthen the network life cycle.

The rest of this paper is organized as follows. Section 2 gives a concise discussion of related schemes for improving the target area and reducing energy consumption in wireless sensor networks. Section 3 defines the system model and defines related terms. Section 4 comes up with our proposed model of 3D-Voronoi and K-means for Energy-efficient Coverage Enhancement (VKECE-3D), with detailed explanations of the algorithms used and their roles at each stage of the algorithm’s operation. Section 5 gives the simulation parameter settings as well as an examination of the simulation experimental data. Section 6 summarizes the work and provides an outlook on the next steps to be taken.

## 2. Related Works

During these years, research on swarm-intelligence algorithms has been in full swing. Some classical swarm-intelligence algorithms are commonly employed to decide coverage enhancement tasks in WSNs. For example, Wang et al. [14] came up with a heuristic algorithm, the Enhanced Sparrow Search Algorithm according to Firefly (EFSSA), to improve the sparrow search algorithm by using an elite inverse solution strategy and the firefly algorithm to avoid the local optimum problem during population search to enhance the network’s coverage quality. The literature [15] proposed the virtual force-embedded Lévy flight grey wolf optimization algorithm (VFLGWO). The phenomenon of premature convergence is solved by introducing the Lévy flight tactics, and the gravitational and repulsive forces between virtual nodes are used to improve the homogeneity of the network, which has strong performance in terms of coverage, the degree of homogeneity, and the average moving distance. Deepa et al. [16] proposed the Lévy light-fused whale optimization algorithm (LWOA). Lévy flight tactics and K-Nearest Neighbor were introduced to balance the optimization finding capability of the algorithm and lower network cost. The algorithm’s efficacy was evaluated in a real-world irrigated agriculture scenario. Chowdhury et al. [17] came up with FIS-RGSO, a reverse firefly swarm optimization-based dynamic fuzzy inference system. Fuzzy inference system decision-making enables restricted and structured sensor movement, which increases the network’s sustainability, and in regard to lifetime and uniformity, the effects are excellent. Yin et al. [18] proposed a hybrid algorithm that combines yin-yang pair optimization and pigeon-inspired optimization algorithms to effectively improve the network coverage quality.

Computational geometry and mathematics are also common methods of coverage enhancement. Based on the positive polyhedron and moment models, Xiao et al. [19] determined the relationship between coverage and sensing radius and computed the minimal quantity of nodes necessary to maintain full network coverage. Farman et al. [20] proposed a novel grid-based hybrid network deployment strategy (GHND) to partition high-density network areas and combine low-density network areas. This strategy ensures the network’s energy efficiency and load balancing. Charr et al. [21] came up with a novel mixed-integer linear programming method (MILP) and designed a dedicated heuristic algorithm (DH) to solve the maximum network lifetime problem (MLP) for HWSNs under connectivity and coverage limitations, which effectively meets the network QoS metrics.

To enhance the network coverage quality, coverage holes in the network may be detected and repaired. For instance, Hao et al. [22] used computational geometry in a 3D surface environment to detect holes step by step and used virtual forces to adjust the node positions according to the movement direction and distance, thus repairing the coverage holes and further ensuring network coverage and effective connectivity. The literature [23] came up with a Multi-Factor Collaborative Hole Repair Optimization Algorithm (FCH-ROA) for HWSNs, which constructs Pearson-like fuzzy matching relationships between mobile nodes and candidate nodes to repair the covered hole area and generates a decision table considering key nodes, remaining energy, and relative distance. Meanwhile, the mobile node moves a limited distance according to the decision table to complete the repair of the coverage holes. Zhang et al. [24] used the concepts of Turan’s theorem, graph theory degree of neutrality, and clustering coefficients to identify redundant nodes. The theory of complete graph is employed to establish the detection and deletion rules of useless edges and to mark the holes in the network from the perspective of loops. The results demonstrate the method’s superior detection efficiency. Le et al. [25] proposed the hole-detection and repair protocol TELPAC, which uses polygons to approximate holes. Based on the approximation, the location to be repaired is found by using regular hexagons. The network time consumption and energy overhead are reduced based on improving network coverage.

Some other researchers have focused on extending the network life cycle. For example, Jia et al. [26] divided the nodes into normal, advanced, and super nodes and respectively gave them different probability selection formulas. Pairing the nodes and sleeping one of them lengthens the network life cycle. However, they need to face high-time complexity problems, which leads to poor network timeliness. Katti et al. [27] came up with a node-scheduling algorithm that minimizes the quantity of active nodes. Reducing the quantity of sensor nodes used in coverage monitoring reduces the deployment cost. Bairagi et al. [28] proposed the multi-objective integer linear programming-based method APP_7 and the non-dominated ranking genetic-based heuristic algorithm APP_8 for the energy-consumption optimization problem of 3D scenes. This approach maintains excellent performance in large and complex environments. Haider Ali et al. [29] proposed an innovative heuristic strategy for selecting cluster heads called ARSH-FATI. Improved network lifetime (LT) and lower transmission energy requirements are two benefits of employing the NRC ranking-based clustering method. With its ability to dynamically transition between exploration and exploitation stages during operation, ARSH-FATI delivers a high degree of load balancing and greatly boosts the LT of the network compared to conventional techniques.

Furthermore, Voronoi diagrams generate an essential influence in research around coverage optimization. Chowdhury et al. [30] proposed an energy-efficient coverage enhancement method for WSNs based on the Tyson Voronoi-Glowworm Optimization K-Means algorithm. With the assistance of the firefly algorithm, the clustering algorithm and Voronoi structure with a minimum of active nodes enhance the network coverage area and use a sleep-wake mechanism to reduce node energy consumption, improving the deployed network lifetime. Dang et al. [31] extended the Voronoi principle to two-dimensional planes and combined the virtual force algorithm to optimize the three-dimensional Voronoi partition. They set the priority coverage mechanism to effectively enhance the target network’s coverage performance. The literature [32] proposed a Repair Optimization Algorithm for Node Stable Matching (ROA-NSM) for coverage hole repair by combining Voronoi polygons and Delaunay triangles to achieve the optimization of coverage hole repair by establishing a stable matching relationship between virtual repair nodes and mobile nodes. Wang et al. [33] used the virtual force between a polygon vertex and its neighbor nodes as a perturbation factor for a cuckoo position update based on the Voronoi partition of the network and completed coverage optimization by using the Virtual Force and Cuckoo Search algorithm VF-CS. Mahboubi et al. [34] came up with tactics to achieve the coverage enhancement of HWSNs using Voronoi diagrams. The multiplicative weighted Voronoi diagram, MW-Voronoi, was used to handle sensor nodes having different sensing radii, but the application scenario of this algorithm has significant limitations.

From the above discussion, we can see that most existing studies focus on increasing coverage or reducing energy consumption without effectively combining coverage increase and energy consumption reduction. We propose a scheme that effectively balances coverage and energy consumption to enhance the coverage and life cycle of the network. First, a secondary deployment strategy is used to improve the homogeneity of the network. Second, K-means is used to determine the optimal location of sensors, and 3D-Voronoi partitioning is employed to determine the optimal sensing radius required to achieve maximum coverage quality. Finally, multi-hop communication and polling working mechanisms are proposed to lower node energy consumption and lengthen the network life cycle.

## 3. System Model and Term Definition

### 3.1. Network Model

It is recommended to presume that all mobile nodes in the network are dormant before attempting to improve coverage. The network can be built as an HWSN [23] by randomly deploying N nodes with various sensing radii in the target area R. The network model in this research is built on the following assumptions.

**Hypothesis** **1.** *In the network, each sensor node has a globally unique identification and can identify its precise location using GPS or other location services*.

**Hypothesis** **2.** *Generally, there are variances in the sensing range of network nodes, but the communication radius*$R_{c}$*is twice the sensing radius of*$R_{s}$*, i.e.,*$R_{c}=2R_{s}$. *The sensing and communication ranges are spherical regions with the node location as the center and with*$R_{s}$, $R_{c}$*as the radius.*

**Hypothesis** **3.** *At least one node in the target area detects the monitoring point due to the random nature of node deployment, proposing that events happening at that location can be efficiently detected*.

**Hypothesis** **4.** *The distributed base station is responsible for waking up the dormant node when the node energy is below the threshold*.

**Hypothesis** **5.** *Sensor nodes, as the smallest IoT devices, have identical energy, calculation, transmission, and communication capability and differ only in sensing range*.

### 3.2. Perceptual Model

The environmental monitoring of HWSNs is achieved through the collaboration of individual nodes. Therefore, the perception models of individual sensor nodes must be modeled in advance in coverage studies of HWSNs. In recent research, Boolean sensing models have been commonly employed to represent the sensing ability of nodes. In this model, a node’s perception probability is 1, indicating that the target is within the node’s perception range, and 0 indicates that it is not covered. The Boolean model has been widely used due to its simplicity and ease of use for experimentation. As a result, in an actual monitoring system, the sensor node sensing probabilities are routinely affected by the environment, signal transmission distance, and other factors. Therefore, this research employs a probabilistic sensing model, which better reflects the characteristics of the node sensing distance [35].

(1) $$P(i,p)=\left\{\begin{array}{ll} 1 & 0\leq d_{i,p}<R_{\min}\\ e^{-\lambda \alpha^{\beta }} & R_{\min}\leq d_{i,p}<R_{\max}\\ 0 & d_{i,p}\geq R_{\max} \end{array}\right.,$$

where $d_{i,p}$ is the distance between nodes i and target points p, measured by Euclidean distance, and $d_{i,p}=\sqrt{{\left(x_{i}-x_{p}\right)}^{2}+{\left(y_{i}-y_{p}\right)}^{2}+{\left(z_{i}-z_{p}\right)}^{2}}$. $R_{max}$, $R_{min}$ indicate the maximum and minimum sensing radius of nodes in the target area, respectively. $\alpha =d_{i,j}-R_{min}$, λ and β are adjustable parameters of sensor node-sensing capability.

### 3.3. Energy Consumption Model

Nodes in HWSNs are commonly powered by non-rechargeable batteries. As a result, the energy problem has a significant influence on the network life cycle. To be able to transmit data efficiently, this research uses the energy consumption model proposed in the literature [36] for energy consumption calculation, which uses part of the node energy for signal amplification based on the node transmission distance. The energy consumed by a node transmitting *k* bits of information via a link could be described as:

(2) $$E_{tx}(k,d)=\left\{\begin{array}{ll} kE_{elec}+k\varepsilon_{fs}d^{2} & \;if\;d<d_{0}\\ kE_{elec}+k\varepsilon_{amp}d^{4} & \;if\;d>d_{0} \end{array},\right.$$

(3) $$d_{0}=\sqrt{\frac{\varepsilon_{fs}}{\varepsilon_{amp}}},$$

where $E_{elec}$ is the quantity of energy required by the node to transmit 1 bit of information, $\varepsilon_{fs}$ is the power amplifier loss coefficient under the free-space model, and $\varepsilon_{amp}$ is the power amplifier loss coefficient under the multipath fading model. The energy consumption equation for a node receiving k bits of information is as follows:

(4) $$E_{rx}(k,d)=k\times E_{elec\;},$$

The moveable distance of a mobile node does not exceed its maximum moveable distance, i.e.,

(5) $$d_{max}=\sqrt[{n}]{\frac{E_{res}}{k}},$$

where $d_{max}$ denotes the node can be moved the maximum possible distance, and $E_{res}$ is the node’s remaining energy.

### 3.4. Description of Connectivity

One of the most fundamental needs for HWSNs is the ability to connect to a network. The connection criterion can only be satisfied if the communication radius $R_{C}$ is larger than or equal to twice the sensing radius $R_{S}$. For proper two-way communication between any two locations i($x_{i},y_{i},z_{i}$) and j($x_{j},y_{j},z_{j}$) in the three-dimensional stereoscopic space, the following requirements must be true.

(6) $$d_{ij}=\sqrt{{\left(x_{i}-x_{j}\right)}^{2}+{\left(y_{i}-y_{j}\right)}^{2}+{\left(z_{i}-z_{j}\right)}^{2}}\leq R_{C},$$

After determining the level of connectivity between any two nodes, the next step is to construct an adjacency matrix $M_{v}$ of the undirected graph, which is created to maintain a record of the connection status between any two nodes. The connectivity is then determined using Equation (7) after the adjacency matrix $M_{v}$ has been populated with the relevant information. If the value of $M_{v}\left[i\right]\left[j\right]$ is 1, it signifies that the i-th node and the j-th node are able to communicate with one another and share information, but a value of 0 indicates that the two nodes are separated from one another. When everything is said and done, the connection of the whole network is analyzed using the matrix power technique in [37]. Calculation of the matrix vector $S_{v}$ is done using Equation (8).

(7) $$M_{v}[i][j]=\left\{\begin{array}{ll} 1 & \;if\;s_{i}\;and\;s_{j}\;can\;communicate\;\\ 0 & \;otherwise\; \end{array}\right.,$$

(8) $$S_{v}=M_{v}+M_{v}^{2}+M_{v}^{3}\cdots +M_{v}^{n-1},$$

If there is an element of $S_{v}$ that is 0, then the network is not linked; if there is not such an element, then the network is connected where n is the quantity of sensor nodes. The Kruskal mechanism [38] is employed in this situation to generate the tree with the minimum spanning branches, which is determined by the connectedness of the HWSNs.

### 3.5. Related Concepts

Sensor nodes $S_{i}\left(i=1,2,3,\ldots ,n\right)$ are deployed randomly in the target area. To enhance network service quality, the uncertainty and complexity of the deployment environment are being considered. If the target area coverage ratio $R_{cov}$ is more than 0.9, it is deemed to have trustworthy coverage quality. Then, the nomenclature and important concepts used in this study are defined in Table 1.

**Definition** **1 (Joint sensing probability).** *Random deployment of many nodes in the target area leads to various sensor nodes sensing the identical event. If the coverage of the sensor node*$S_{i}$*to point*p*is*$s_{p}\left(s_{1}\right),s_{p}\left(s_{2}\right),\ldots ,s_{p}\left(s_{n}\right)$, *the joint perception probability of target point*p*in the network is:*

(9) $$P_{p}(R)=1-\prod_{i=1}^{N}\left[1-p(i,p)\right],$$

**Definition** **2 (Coverage).** *The coverage of the whole network could be determined by partitioning the network into* k *discrete points and averaging the coverage of these discrete points:*

(10) $$R_{\mathrm{cov}}=\frac{\sum_{i=1}^{k}P_{i}(R)}{k},$$

*where* $P\left(R\right)$ *is the statistical value in the simulated environment. If the value of* k *is large enough, then the simulated environment value and the actual value are infinitely close to each other.*

**Theorem** **1.** *Based on the study in the literature [30], it is known that in the two-dimensional plane environment where the sensor-node sensing area is circular, the area of the sensing circle can cover the whole Voronoi diagram when the farthest distance from the center of mass to the vertex of the Voronoi diagram is employed as the optimal sensing radius. Extending this to the three-dimensional environment, the volume of the perception sphere will cover the whole cell when the farthest vertex from the center of mass to the 3D-Voronoi cell is chosen as the perception radius*.

**Figure 2:**
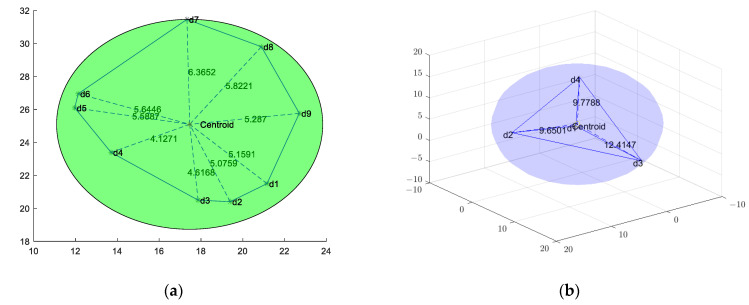
Schematic of Voronoi’s optimal sensing radius coverage: (**a**) Two-dimensional case; (**b**) Three-dimensional case.

A schematic of two-dimensional and three-dimensional Voronoi is shown in Figure 2. In Figure 2a we see that the distance from $d_{7}$ to the center of mass is the farthest at 6.3652 and using it as a radius to draw a circle can cover the whole graph Figure 2b shows that the distance from $d_{3}$ to the center of mass is the farthest at 12.4147, and using it as a radius to draw a ball can cover the whole area.

## 4. Energy-Efficient Coverage Enhancement Algorithm VKECE-3D

### 4.1. Highly Destructive Polynomial Mutation Strategy

After the airdrop of HWSN nodes, the target area has problems with high aggregation leading to low coverage, which may have an impact on network service quality. Mutation theory was proposed in 1972 and originally meant an interruption or abrupt transition of the process of development. In recent HWSN research, this technique has been employed to improve the algorithm’s capacity to break out of the local optimal solution. Based on the literature [39], we introduce a highly destructive polynomial mutation strategy for the secondary deployment of nodes in this research to enhance node uniformity. It is like the mutation operation of the genetic algorithm, chaos theory, Lévy flight perturbation strategy, etc. Its main advantage is that the whole search range of decision variables can be explored even if the current search value comes close to the search space constraints. Therefore, the introduction of the highly destructive polynomial mutation strategy serves to further enhance the network coverage and lay the foundation for the next clustering algorithm.

(11) $$\delta_{k}=\left\{\begin{array}{ll} {\left[2r+(1-2r)\times {\left(1-\frac{x_{i}-lb}{ub-lb}\right)}^{\eta +1}\right]}^{\frac{-\eta }{\eta +1}} & \;if\;r\leq 0.5\\ 1-{\left[2(1-r)+2(r-0.5)\times {\left(1-\frac{ub-x_{i}}{ub-lb}\right)}^{\eta +1}\right]}^{\frac{1}{\eta +1}} & \;otherwise\; \end{array}\right.,$$

(12) $$x_{i}=x_{i}+\delta_{k}\times (ub-lb),$$

where r is a random variable between [0, 1], lb and ub are the lower and upper boundaries of the target space, $x_{i}$ is the coordinates of the sensor node, and η is a non-negative value for the distribution index.

**Figure 3:**
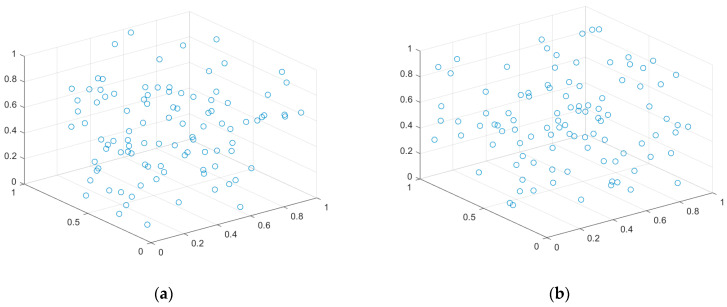
Network uniformity effect diagram: (**a**) Chaotic mapping; (**b**) Highly destructive polynomial mutation strategy.

The results of randomly deploying 100 sensor nodes in the [0, 1] search space, utilizing chaotic mapping and highly destructive polynomial mutation strategy, are depicted in Figure 3. The enhanced homogeneity of the network nodes is seen in Figure 3b, as opposed to the chaos theory impact in Figure 3a.

In the network, when the location of nodes mutates throughout the process, the position of the mutated node may extend beyond the boundaries of the search area. For most researchers, the present situation is extremely simple. Node locations that change outside of the search space are immediately re-updated to the nearest area border. Although this strategy can minimize algorithm complexity, it also has the disadvantage of diminishing node homogeneity. As a result, the following equation is used in this research to represent a unique mutation crossing processing approach:

(13) $$x’=\left\{\begin{array}{cc} lb+\mu (ub-lb) & \mu <\sigma ,x<lb\\ lb & \mu >1-\sigma ,x<lb\\ ub-\mu (ub-lb) & \mu <\sigma ,x>ub\\ ub & \mu >1-\sigma ,x>ub \end{array}\right.,$$

where x′ is the new location after the mutation crossing node has been updated, μ  is a random value between [0, 1], and σ is the mutation crossing processing factor, which is commonly set to 0.5.

When the node position mutation in the network is completed, the original position and the mutation position are merged and sorted, and the top N nodes with higher network coverage are selected using the greedy strategy, which becomes the input of the next stage algorithm.

### 4.2. K-Means Clustering

Clustering is the classification and organization of data members in a dataset that is similar in certain ways. The most well-known technique for partitioning clusters is K-means clustering, and due to its simplicity and effectiveness, it is the most routinely employed clustering algorithm [40].

The K-means algorithm’s inputs are the sample data set and the quantity of cluster *k*. The output is the k-divided clusters. Its detailed steps are as follows:

1. Select k sample data points at random from the sample data set as the primary cluster center $a=a_{1},a_{2},a_{3},\ldots ,a_{k}$;
2. The Euclidean distance is used to compute the distance between the sample data $x_{i}$ and the center of each cluster, and the clusters with the smallest distance between them are partitioned;
3. After one round of division, recalculate the cluster center of each cluster $a_{j}$ using Formula (14);
  (14) $$a_{j}=\frac{1}{\left|C_{i}\right|}\sum_{x\in C_{i}}x,$$
4. Repeat the above two operations until the partition result is unchanged.

The quantity of clusters and the initial cluster centers need to be determined before using K-means clustering. Therefore, to apply it to the HWSNs scenario, the initial cluster centers and the quantity of clusters should first be determined. In this research, the initial clustering centers are determined by the random initialization method—the core indicator for confirming the quantity of clusters using the elbow method is the sum of the squared errors (*SSE*), i.e.,

(15) $$SSE=\sum_{i=1}^{k}\sum_{x\in C_{i}}{\left|x-m_{i}\right|}^{2},$$

where $C_{i}$ is the i-th cluster, x is the sample data in it, and $m_{i}$ is the mean data of all samples in $C_{i}$, i.e., the $C_{i}$ cluster center. The clustering error of all samples is defined as *SSE*, and its magnitude measures the quality of cluster delineation.

With the increase of the quantity of clusters k, the granularity of the sample partition becomes finer, and the *SSE* will gradually go down as the degree of cluster cohesiveness rises. If k is smaller than the real clustering quantity, the degree of cluster cohesion changes dramatically with the increase of k, so the decreasing trend of *SSE* will be obvious; when k is equal to the quantity of real clustering, the degree of cluster cohesion decreases rapidly with the increase of k, so the decreasing trend of *SSE* will abruptly decrease. When k continues to increase while *SSE* tends to level off, the changing relationship between *SSE* and k is like the shape of an elbow. Therefore, it is called the elbow method, and the k corresponding to the elbow is the real quantity of clusters required for the sample [41].

For the coverage optimization scenario under HWSNs, we chose K-means to divide the nodes into many clusters after secondary deployment, randomly select the initial centers of mass, and guide the algorithm to solve the local optimal partitioning scheme. To a certain extent, the K-means clustering algorithm’s accuracy is determined by the initial clustering center, and its low time complexity is also a benefit when compared to other clustering algorithms. The K-means algorithm determines that cluster centroids are the ideal locations for node deployment.

### 4.3. 3D-Voronoi Partition

In practice, the target area cannot be completely covered owing to the initial deployment environment being complex, or the hole is generated sensor-node offline, energy exhaustion, and so on. Nodes in HWSNs cannot receive information from the target area—possibly affecting the quality of the customer experience. Therefore, further sensor node deployment may improve the network’s coverage. In a two-dimensional environment, coverage holes may often be discovered using Voronoi diagrams. Then, the network’s whole target area is partitioned into sections using the Voronoi cell principle, and the vertical bisector of the line connecting the points in two regions is the boundary line of the Voronoi diagram.

Based on 2D-Voronoi, it is extended to the 3D environment for partitioning. The three-dimensional space is divided into Voronoi polyhedral cells, where each cell is an irregular, polyhedral, closed convex body, which is defined as a 3D-Voronoi cell. To improve coverage quality while decreasing energy consumption, the originally deployed sensors are moved, and the detecting range of each sensor node is adaptively modified, employing 3D-Voronoi cells. All of the 3D-Voronoi cells use the K-means algorithm’s cluster centers as their centroid points.

Suppose there are k centers of mass $s_{k}=\left\{s_{1},s_{2},\ldots ,s_{k}\right\}$ in the space L×L×L, which are divided into k cells, $V_{k}=\left\{V_{1},V_{2},\cdot \cdot \cdot ,V_{k}\right\}$. The cell $V_{i}\left(i=1,\;2,\cdot \cdot \cdot \cdot ,k\right)$ comprises a one-of-a-kind center of mass $c_{i}$, then $c_{i}$ is defined as the generating location of $V_{i}$ [42]. Thus, the distance from whatever location p to the center of mass $c_{i}$ within a given cell $V_{i}$ is shorter than the distance from p to the additional $c_{j}$. The 3D-Voronoi structure is described as continuing to follow:

(16) $$Q\left(V,V_{i}\right)=\left\{V_{i}\in V|d\left(p,\left(V,c_{i}\right)\right)\leq d\left(p,\left(V,c_{j}\right)\right),j=1,2,\ldots ,k\right\},\forall j\neq i,$$

The distance from a location $p_{i}\left(x_{i},y_{i},z_{i}\right)$ in the unitary cell to any center of mass $c_{j}\left(x_{j},y_{j},z_{j}\right)$ in space is:

(17) $$d\left(p_{i},c_{j}\right)=\sqrt{{\left(x_{i}-x_{j}\right)}^{2}+{\left(y_{i}-y_{j}\right)}^{2}+{\left(z_{i}-z_{j}\right)}^{2}},$$

Based on the above partition and Theorem 1, we can obtain the following conclusions:

1. The distance from each center of mass to its 3D-Voronoi vertex is calculated, and the cell’s optimal perceptual radius is confirmed by the greatest distance between them.
2. Quantity of 3D-Voronoi cells is equal to the minimum quantity of active nodes needed by the network to achieve the QoS standards. The best deployment position of the active node is defined as the cell’s centroid in 3D-Voronoi space.

**Figure 4:**
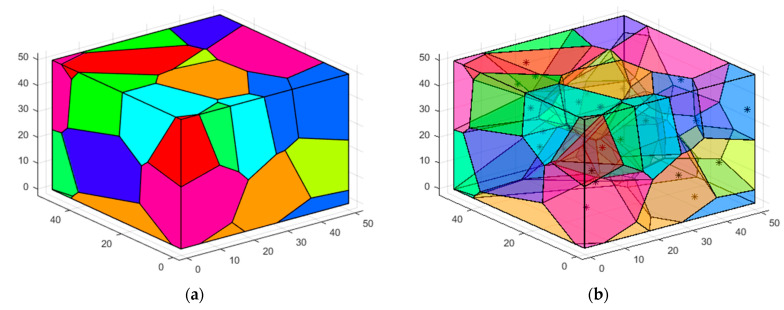
3D-Voronoi partition process diagram: (**a**) Original 3D-Voronoi partition; (**b**) Display centroid 3D-Voronoi partition.

Therefore, the target region is completely covered if all 3D-Voronoi structure vertices are covered. Then, the 3D-Voronoi partitioning process is shown in Figure 4, where * represents the centroid.

### 4.4. Multi-Hop Communication and Polling Working Mechanism

**Figure 5:**
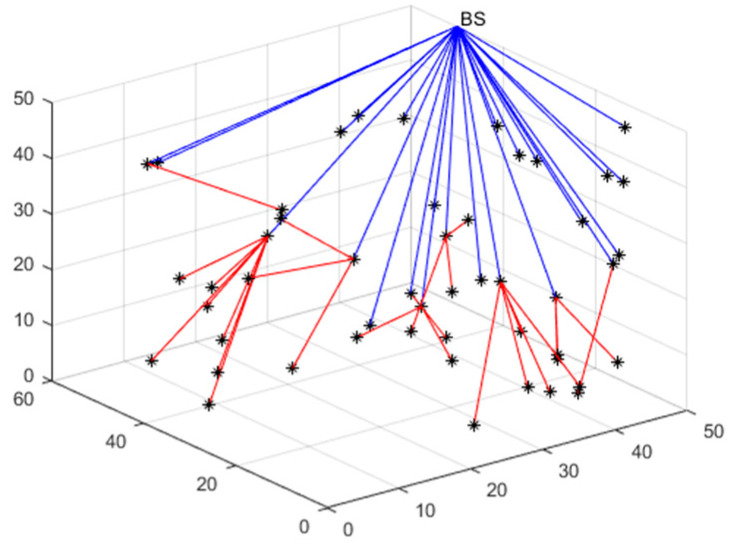
Multi-hop communication mechanism.

Multi-hop communication techniques have several advantages that include reduced interference, increased spectrum reuse, and reduced energy consumption. Among the currently proposed techniques, multi-hop mechanisms have been used for data communication [36]. Figure 5 is used as an example to display the principle of multi-hop communication.

Based on the energy consumption model created by Equation (2), the distributed base station is positioned in the network’s top right corner, and the single-hop to multi-hop communication conversion threshold $d_{0}$ is defined at 50 m. The communication process of the network is defined into two categories. If the node’s distance from the distributed base station is less than a specific threshold, the node can establish a direct communication link with the distributed base station and send data through this link. Otherwise, the node must first establish a link with a node near the distributed base station, after which it must send the data to an intermediate node, which finally transmits it to the distributed base station. Due to the additional effort required to coordinate the involvement of several nodes, multi-hop communication uses more energy than its single-hop version.

In Figure 5, nodes using multi-hop communication are marked by red paths. The communication process is established as follows. Firstly, the node that needs to communicate establishes communication with its single-hop neighbors, and blue paths indicate intermediate forwarding nodes with enough energy. Secondly, the intermediate nodes gradually communicate data until they establish a stable link with the base station.

Energy consumption is an enduring hot issue in the research field of HWSNs. To lower the communication energy consumption between nodes, this research proposes multi-hop communication and polling working mechanisms. The network nodes’ states are classified as active, dormant, and dead. After completing the 3D-Voronoi division, each unit sorts the nodes according to the distance from the centroid from small to large and determines it the polling working order. It is guaranteed that only one node in each cell is active, and the remaining nodes are in a dormant condition. It is assumed that the nodes’ energy consumption in the dormant state is negligible. When the currently active node is working normally, its optimal sensing radius guarantees a high coverage of the current unit. Assume that the node’s initial energy is defined as 5 J. Due to the normal operation of the network, communication links are continuously established between the nodes to exchange data. As the network runs, the quantity of iteration rounds increases while the energy of the active nodes decreases. When the active node energy falls below a predetermined death threshold of 0.02 J, its state is modified to death. The distributed base station follows the pre-established polling work order, and the next node within this cell will be activated and moved to the center of mass to start working until all nodes in a cell are in the death state. With this mechanism, not only is coverage guaranteed, but the network life cycle is also extended as much as possible.

### 4.5. VKECE-3D Algorithm

Using the above theoretical analysis and algorithm description, we develop a way for improving coverage in 3D HWSNs using 3D-Voronoi partitioning and K-means while minimizing energy consumption. We call this approach VKECE-3D. To begin, sensor nodes in the target area are first set up by a random deployment approach, and then they are deployed twice with a highly damaging polynomial mutation mechanism. After selecting the clustering algorithm hyperparameter k using the elbow approach, the sensor network begins executing the suggested K-means and 3D-Voronoi partitioning to reduce the quantity of active nodes, determine the optimum sensing radius, and set up communication between the individual nodes. The energy requirements of active nodes may be reduced, and the lifetime of the network can be extended thanks to multi-hop communication and polling working mechanisms. In light of the above analysis, this section illustrates the logic and procedure of the VKECE-3D algorithm developed in this research.

Step 1 Initialization phase: In the target area R, a random deployment method is taken to initialize N sensor nodes.

Step 2 Secondary deployment: Using the highly destructive polynomial mutation strategy, Equations (11), (12), the nodes are deployed twice and can be relocated using Equation (13) if the nodes mutate beyond the search space.

Step 3 Clustering: The quantity of clusters k is determined using the elbow method. Cluster the nodes using K-means and find the cluster centers.

Step 4 3D-Voronoi partitioning: Based on the clustering centers satisfying Equation (16), a 3D-Voronoi partition is executed to partition the network into cells of k.

Step 5 Calculate the optimal perception radius: To establish a perceptually heterogeneous wireless sensor network, the ideal sensing radius of each cell is determined by calculating the farthest point from the cell’s center to the vertex of the 3D-Voronoi cell.

Step 6 Find the ideal location to work in: Within each 3D-Voronoi cell, the node closest to the center of mass moves to the center of mass and starts working, while the rest of the nodes are dormant.

Step 7 Checking connectivity and calculating coverage: In order to ensure that no disconnected nodes exist in the network, Equations (7), (8) are used, and Kruskal’s method is used to create the shortest possible tree topology. Calculate the network coverage using Equation (10). Repeat Steps 3–7 until the coverage of the network, $R_{cov}$, is more than 90% and the conditions are met.

Step 8 Multi-hop communication and polling working mechanisms: After the death of the node in the current cell, the next node nearest to the center of mass that is in dormant mode is activated and moves to the center of mass position to continue working.

Algorithm 1 gives the pseudo code of the VKECE-3D algorithm.

**Algorithm 1**: VKECE-3D**Input**: Location of N sensor nodes; Maximum number of iterations $T_{max}$ **Output**: Deployment Solutions 1: In the target area R, a random deployment method is taken to initialize N sensor nodes. 2: Secondary deployment using highly destructive polynomial mutation strategy nodes 3: **for** (t = 1, 2…,$T_{max}$) 4: Determine the clustering algorithm hyperparameter k  using the elbow method 5: Cluster the nodes using K-means and find the clustering centers 6: Perform 3D-Voronoi partitioning based on clustering centers 7: The optimal perceptual radius of a cell is defined as the farthest distance from the centroid to the vertex of the 3D-Voronoi cell 8: Within each 3D-Voronoi cell, the node closest to the center of mass moves to the center of mass and starts working, while the rest of the nodes are dormant 9: Verify network connection, Kruskal’s algorithm constructs a minimal spanning tree 10: If the coverage of the network $R_{cov}$ is higher than 90% and the connection of the network satisfies the criteria, stop the loop; otherwise, continue 11: Return best deployment solutions 12: The multi-hop communication and polling working mechanisms start to take effect.

**Figure 6:**
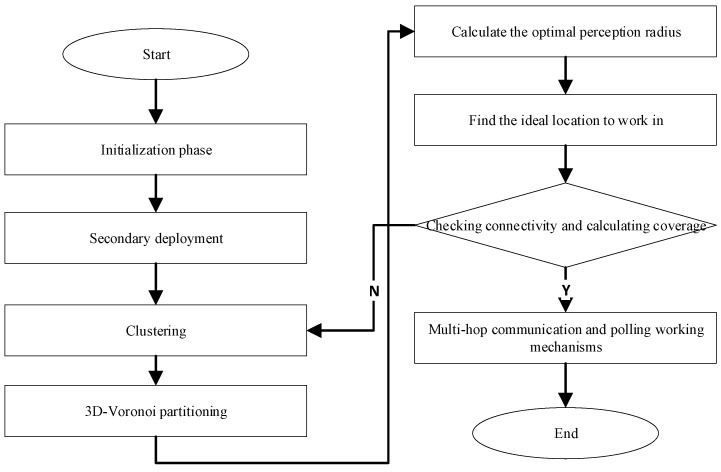
VKECE-3D algorithm flowchart.

The flow of the VKECE-3D algorithm is shown in Figure 6.

### 4.6. Complexity Analysis

For the VKECE-3D algorithm, N is the quantity of mobile nodes, T is the maximum quantity of iterations, and k is the quantity of clusters. The time complexity of the highly destructive polynomial mutation strategy is $O\left(N\right)$, the time complexity of K-means clustering is $O\left(k\times N\right)$, the time complexity of 3D-Voronoi partitioning and calculating the optimal sensing radius is $O\left(k\times N\right)$, and the time complexity of the multi-hop communication and polling working mechanism is $O\left(N\right)$. Thus, the total time complexity is $O\left(2NT+2kNT\right)$, which is reduced to $O\left(NT\right)$, since T ≫ k.

## 5. Simulation Result Analysis

### 5.1. Experimental Environment

To verify the effectiveness and performance of the algorithm, we perform simulation experiments using MATLAB 2020a, all on Windows 11, using an Intel i5-7200U Core 2.71 GHz CPU, and 16 G RAM. VKECE-3D is compared with GHND [20], CSPM [39], CS [43], MACHPS [13], and IPSO-IRCD [44]; and the experiments are divided into two parts: simulation runtime and performance testing. We deploy 100, 250, 500 mobile nodes randomly in 50 × 50 × 50 $m^{3}$, 200 × 200 × 200 $m^{3}$, 600 × 600 × 600 $m^{3}$ cube. The specific simulation parameters in the experiments are shown in Table 2.

### 5.2. Method of Comparison

To verify the performance of VKECE-3D, we chose the following five types of comparison methods:

1. GHND [20]: GHND (Grid-based Hybrid Network Deployment) is a representative algorithm to ensure energy-efficient deployment schemes based on grid hybrid deployment to upgrade the energy efficiency and load balancing of WSNs. In the network, a technique of splitting and merging is proposed to enhance the uniformity of sensors. The network can enhance the best load balance state by merging the low-density adjacent areas and splitting the high-density adjacent areas. Benefits in load balancing, network life cycle, and efficiency are all high when using this strategy.
2. CSPM [39]: CSPM (Cuckoo Search with highly disruptive Polynomial Mutation) is initially designed to improve the problem of scheduling algorithms in cloud computing environments tending to fall into local optima. Its application to WSN node deployments improves node uniformity and thus the coverage of the network.
3. CS [43]: CS (Cuckoo Search Algorithm) is a representative of a traditional swarm-intelligence algorithm to deal with 3D problems in ascending dimensions and is a bionic optimization algorithm that simulates the reproductive characteristics of the aggressive nest-seeking and egg-hatching of cuckoos. It has the advantages of setting fewer parameters, a simple process, and fast convergence and has good robustness for many optimization problems. It has been widely used in industrial design and in other practical problems.
4. MACHPS [13]: An example of a swarm intelligence algorithm combined with a sleep-wake mechanism is MACHPS (Mobile Assisted Coverage Hole Patching Scheme). It has the potential to vastly improve network coverage and extend the useful life of a network. Before anything else, a scattershot network of sensor nodes is placed across the region of interest and left there to remain motionless or in a sleep state. Second, we partition the whole network into squares called “grids,” and we calculate the coverage percentage for each grid. As a candidate grid, we choose the one with the lowest coverage. Particle swarm optimization (PSO) is then utilized to estimate the mobile locations of sensor nodes, waking up dormant mobile sensors to fill in the coverage hole.
5. IPSO-IRCD [44]: IPSO-IRCD is a two-stage coverage optimization strategy that addresses an enhanced particle swarm algorithm with a node coverage schedule. In order to discover potential node placements, IPSO computes the particle fitness value and compares it to the prior ideal value, so avoiding the issue of losing the optimal solution of the original method. To find the best fit between nodes and candidate sites without compromising network coverage, IRCD, an optimization method, takes into account both the coverage increments and the move distance to make its selections. It is a great solution for wireless sensor networks’ coverage redundancy and hole problems.

### 5.3. Operation Process Simulation

To verify the validity and scalability of this research method, we divided VKECE-3D into four cases, running in small, medium, and large networks, respectively.

#### 5.3.1. Case 1: Highly Destructive Polynomial Mutation Strategy for the Secondary Deployment of Nodes

**Figure 7:**
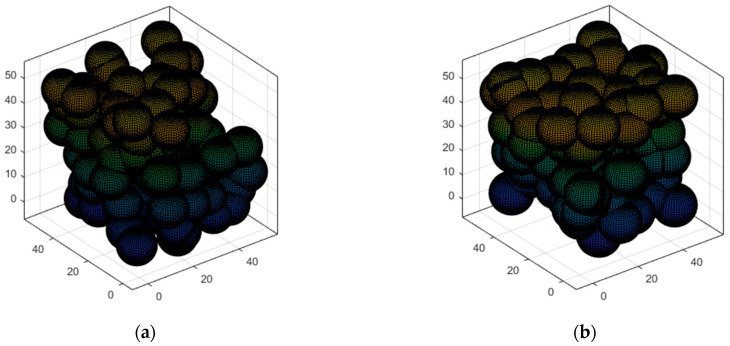

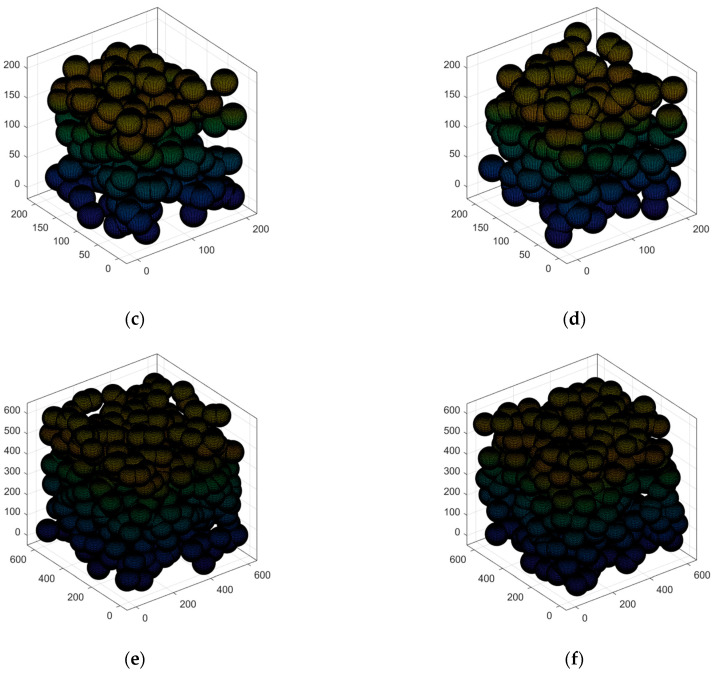
Original and secondary deployment diagrams: (**a**) Random deployment of nodes effect diagram of the small network; (**b**) Highly destructive polynomial mutation strategy effect diagram of the small network; (**c**) Random deployment of nodes effect diagram of the medium-sized network; (**d**) Highly destructive polynomial mutation strategy effect diagram of the medium-sized network; (**e**) Random deployment of nodes effect diagram of the large network; (**f**) Highly destructive polynomial mutation strategy effect diagram of the large network.

In this case, network homogeneity is improved by randomly deploying sensor nodes to simulate the nodes’ movement while the algorithm executes. Figure 7 simulates the process of the secondary deployment of nodes using a highly destructive polynomial mutation strategy. Figure 7a shows the initial, randomly distributed node location map of the small network, which shows the high degree of node aggregation and low coverage—the network currently receives only 68.6% coverage. Figure 7b is a node-location distribution after the secondary deployment of the small network, which can be seen to have improved uniformity compared with Figure 7a, with a coverage rate of 77.8%. However, there is still the problem of high node redundancy. Meanwhile, the node position distribution for the medium-sized network is shown in Figure 7c,d, and the node location distribution for the large network is depicted in Figure 7e,f. The initial coverage of nodes is already larger for medium and large networks, but the redundancy level is also higher. Because of this, the highly destructive polynomial mutation strategy performs well in both moderate and large networks.

#### 5.3.2. Case 2: K-Means Clustering to Determine the Minimum Quantity of Nodes to Be Deployed in the Network

**Figure 8:**
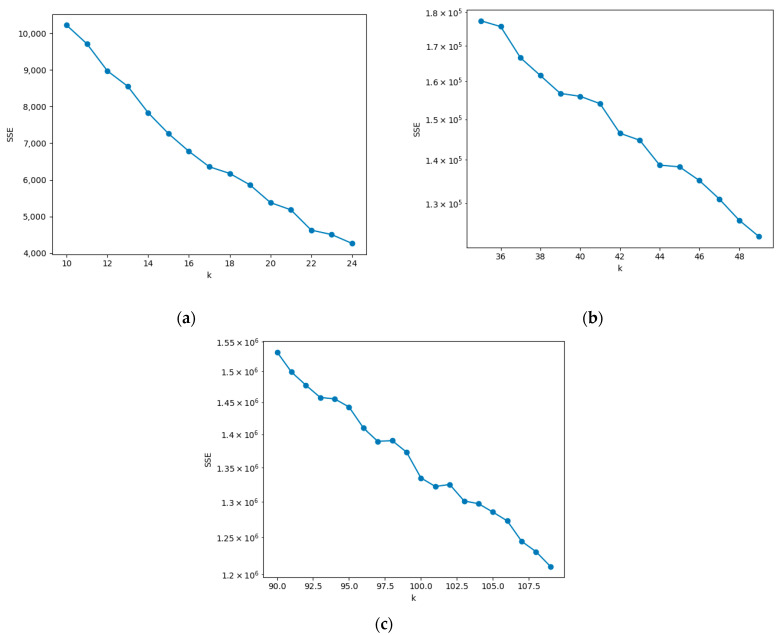
The Elbow method determines the clustering hyperparameter k: (**a**) the small network; (**b**) the medium-sized network; (**c**) the large networks.

**Figure 9:**
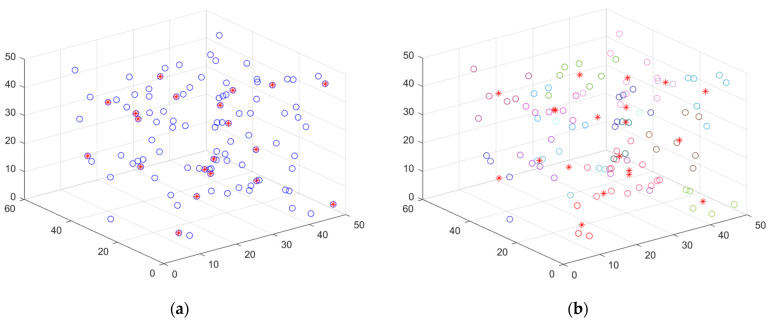

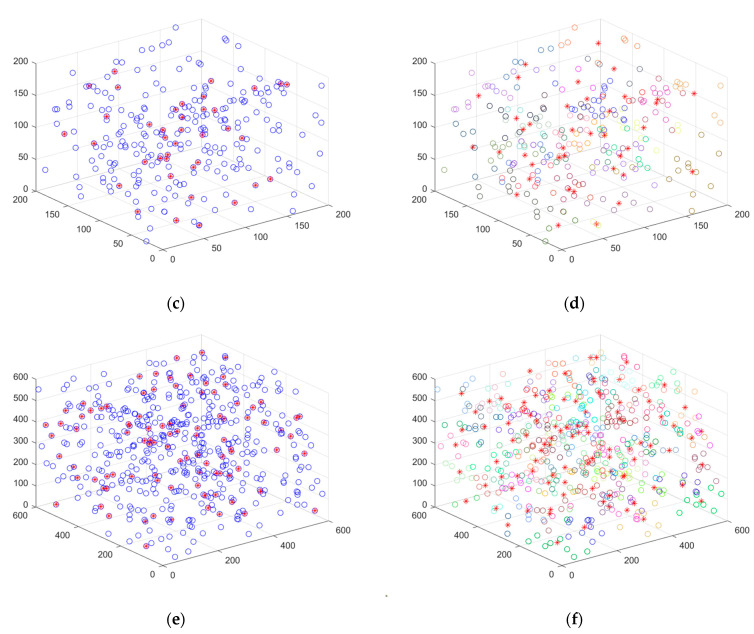
K-means clustering process: (**a**) The small networks: clustering raw data and the center of mass; (**b**) The small networks: K-means clustering results; (**c**) The medium-sized networks: clustering raw data and the center of mass; (**d**) The medium-sized networks: K-means clustering results; (**e**) The large networks: clustering raw data and the center of mass; (**f**) The large networks: K-means clustering results.

After the second wave of nodes has been deployed, they are partitioned into k clusters. This case simulates the selection of the quantity of clusters for clustering and the operation process of K-means. In small networks, it is obvious from Figure 8a that the decline of *SSE* starts to level off when k is at 17, and so k=17 is selected for clustering. And in the medium-sized and large networks, as seen in Figure 8b,c, k=39, 96 were chosen for clustering. The quantity of clusters for clustering at this point is the minimum quantity of nodes deployed in the network. Figure 9 displays the K-means clustering algorithm in action, where nodes of the same hue belong to the same cluster. In Case 2, the red * symbol in the center of the K-means clusters represents the center of mass of the 3D-Voronoi cell. Figure 9a,b shows the results of running a small network, Figure 9c,d shows the results of running a medium-sized network, and Figure 9e,f shows the results of running a large network. In Figure 9, the small circles represent the sensor nodes and * represents the clustering center.

#### 5.3.3. Case 3: Moving the Sensor Nodes to the Optimal Position and Verifying Network Connectivity

**Figure 10:**
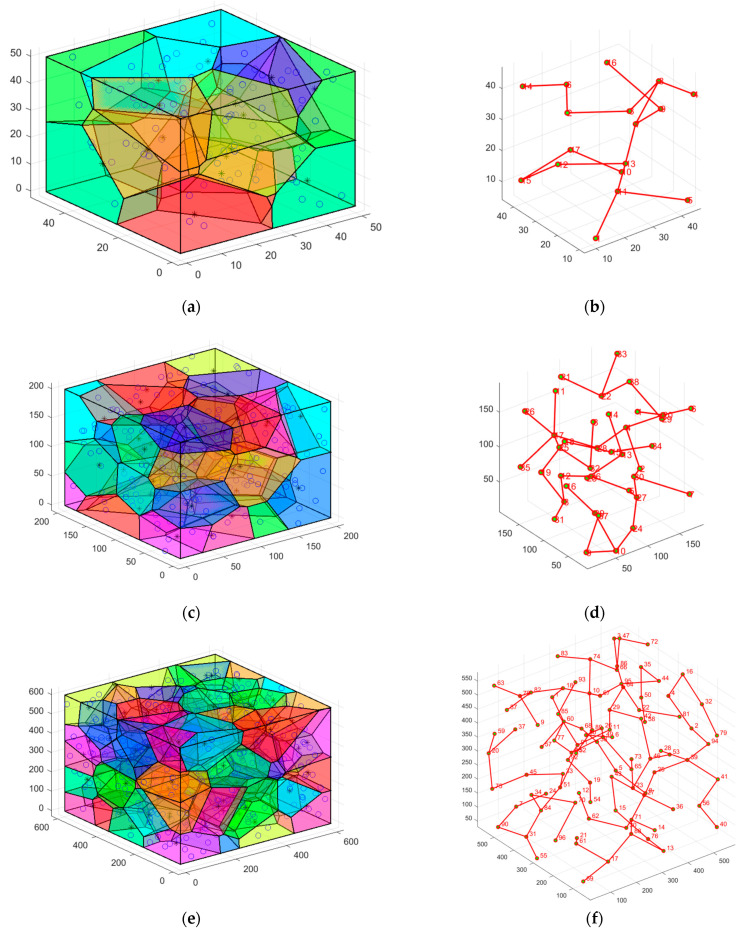
3D-Voronoi partition results and minimal spanning tree: (**a**) The small networks: 3D-Voronoi partition results and center of mass; (**b**) The small networks: minimal spanning tree; (**c**) The medium-sized networks: 3D-Voronoi partition results and center of mass; (**d**) The medium-sized networks: minimal spanning tree; (**e**) The large networks: 3D-Voronoi partition results and center of mass; (**f**) The large networks: minimal spanning tree.

The nodes in Case 2 are divided into 3D-Voronoi by clusters based on the clustering center, the nodes in the same cluster are divided into the same 3D-Voronoi cell, and the position of the center of mass is considered as the best position for the sensor node. Each node in the cluster is dormant except for the one closest to the center of mass, which is chosen and sent there to begin processing information. Figure 10a simulates the 3D-Voronoi partitioning process with * as its center of mass. Theorem 1 guarantees the high coverage of the network, and the algorithm runs to this point. The coverage of the network needs to be calculated. If the threshold of 90% is reached, then it is time to verify the network connection. Equation (8)’s $S_{v}$ contains 0 is utilized to ensure network connection. Next, as seen in Figure 10b, the Kruskal method is used to produce the minimal spanning tree. If those two conditions hold, then Case 4′s polling working mechanism is triggered, and regular operation mode may be carried out. In the alternative, we will proceed with Cases 2–3 until the requirements are met. Figure 10a,b shows the results of running a small network, Figure 10c,d shows the results of running a medium-sized network, and Figure 10e,f shows the results of running a large network. In Figure 10, the small circles represent the sensor nodes and * represents the center of mass.

#### 5.3.4. Case 4: Multi-Hop Communication and Polling Working Mechanism to Lengthen the Network’s Life Cycle

**Figure 11:**
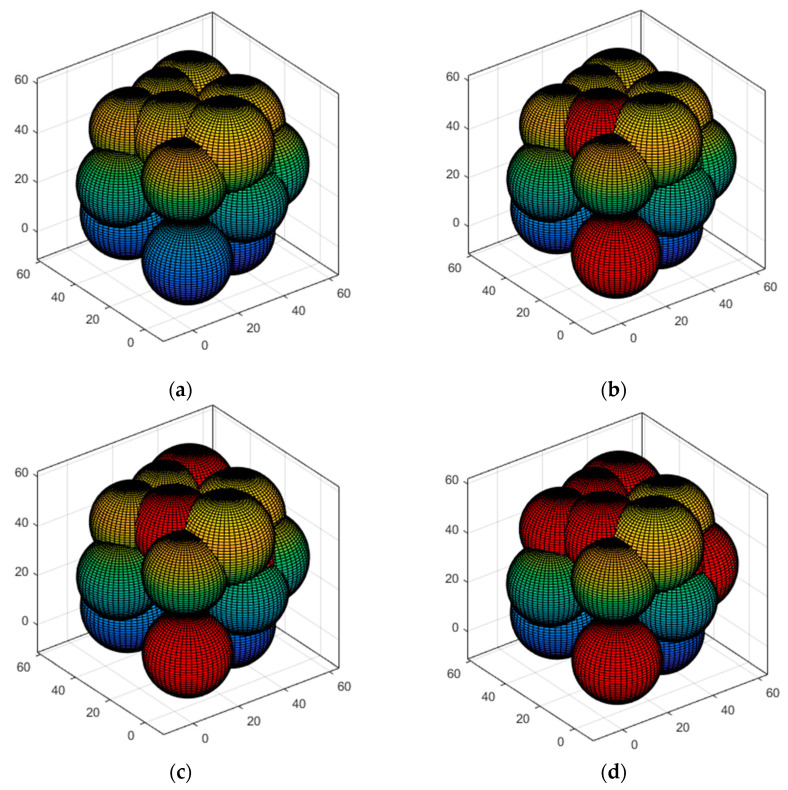
Different rounds of coverage in small networks: (**a**) Network runs 50 rounds; (**b**) Network runs 5000 rounds; (**c**) Network runs 10,000 rounds; (**d**) Network runs 15,000 rounds.

**Figure 12:**
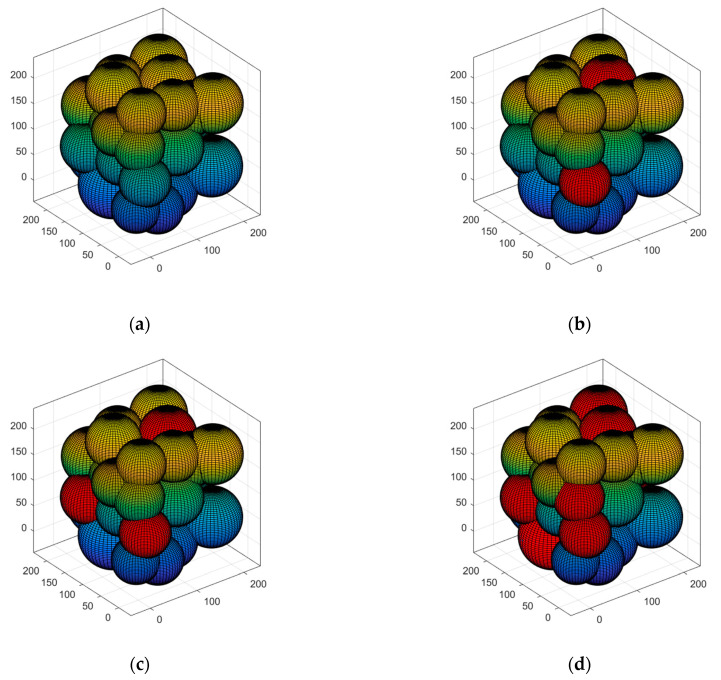
Different rounds of coverage in medium-sized networks: (**a**) Network runs 50 rounds; (**b**) Network runs 5000 rounds; (**c**) Network runs 10,000 rounds; (**d**) Network runs 15,000 rounds.

**Figure 13:**
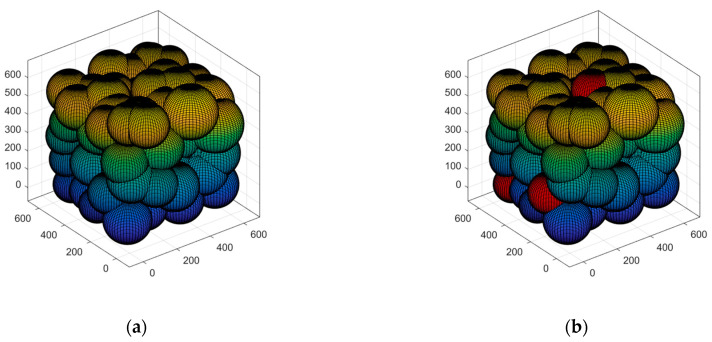

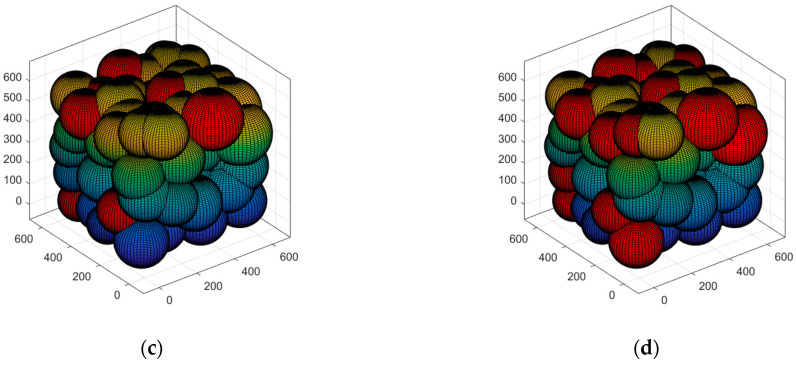
Different rounds of coverage in large networks: (**a**) Network runs 50 rounds; (**b**) Network runs 5000 rounds; (**c**) Network runs 10,000 rounds; (**d**) Network runs 15,000 rounds.

In this case, the algorithm is performed to demonstrate coverage in order to fulfill the network monitoring requirements, which is to simulate the effect of the multi-hop communication and the polling working mechanism proposed by the algorithm in this research, where the color and red represent the covered and uncovered sensing areas, respectively. If at least one node in the cell is active, the cell is covered. As seen in the simulation in Figure 11, the coverage area gradually decreases as the quantity of rounds of the network operation increases. The proposed solution reduces energy consumption by using multi-hop communication and polling primary working mechanisms, although this has a relatively gradual effect on the quantity of active nodes. In this way, coverage may be maintained for a longer time without a progressive reduction in quality. According to Figure 11, the quantity of nodes deployed is 100 in a target area of 50 × 50 × 50 $m^{3}$. The nodes’ initial energy is 5 J. In the whole network, the quantity of nodes that are active at the same time is 17. During the simulation, only 17% of the nodes were active, up to 15,000 rounds. At this point, the coverage rate has dropped to about 53%, indicating that multi-hop communication and polling working mechanisms can extend the network’s life cycle. We then deployed an additional 250 nodes at random in a medium-sized network cube area of 200 × 200 × 200 $m^{3}$, and the simulated results are shown in Figure 12; the approximate number of active nodes is 39. When the network is subjected to 15,000 cycles, coverage falls to about 50%. The simulation results are displayed in Figure 13; the number of active nodes is estimated to be about 96 in a large network cube size of 600 × 600 × 600 $m^{3}$. When the network reaches roughly 15,000 rounds, the coverage drops to about 45%. Overall, the multi-hop communication and polling working mechanisms perform well in all three sizes of networks: small, medium, and large.

Cases 1–4 simulate the running process of VKECE-3D, explain the reasons for the high coverage rate and long-life cycle of the algorithm in graph form, and prove the effectiveness of the method. Detailed performance tests of the algorithm when it comes to the coverage rate, percentage of dead nodes, and percentage of active nodes are described below.

### 5.4. Performance Testing

#### 5.4.1. Coverage Performance Testing

**Figure 14:**
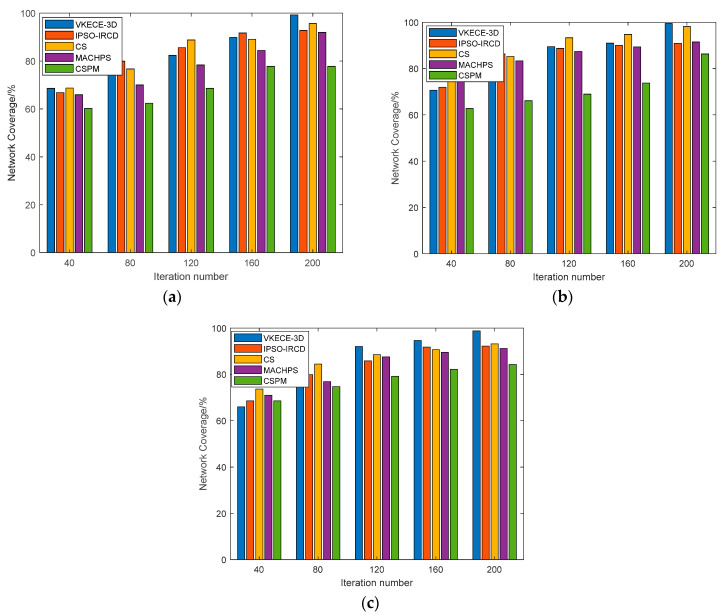
Coverage of our proposed algorithm vs. comparison algorithms: (**a**) the small network, N = 100; (**b**) the medium-sized network, N = 250; (**c**) the large networks, N = 500.

Figure 14 displays the relationship between the network coverage and the number of iterations for the five coverage existing approaches after random deployment of sensor nodes and repeated trials under the same circumstances. According to Figure 14, when more iterations are applied, all five algorithms enhance their network coverage. VKECE-3D can optimize the area coverage from the initial 68.6% to 99.3%, which is an improvement of 30.7%, while IPSO-IRCD, CS, MACHPS, and CSPM improve 26.001%, 26.899%, 26.018%, and 17.6%, respectively. VKECE-3D improves 6.475%, 3.662%, 7.322%, and 13.1% compared with IPSO-IRCD, CS, MACHPS, and CSPM, respectively, indicating that VKECE-3D has a better coverage effect when used for the coverage optimization of 3D HWSNs. Even in medium-sized and large networks, VKECE-3D still maintains good results. However, in the comparison method, as the number of deployed nodes increases, the problem arising from node redundancy becomes more and more prominent, and the coverage of the network improves very slowly. Moreover, the optimal sensing radius proposed in this research stacks the 3D space and ensures the high coverage of the network. Therefore, under the same conditions, VKECE-3D has higher coverage.

#### 5.4.2. Proportion of Dead Nodes in Different Rounds

**Figure 15:**
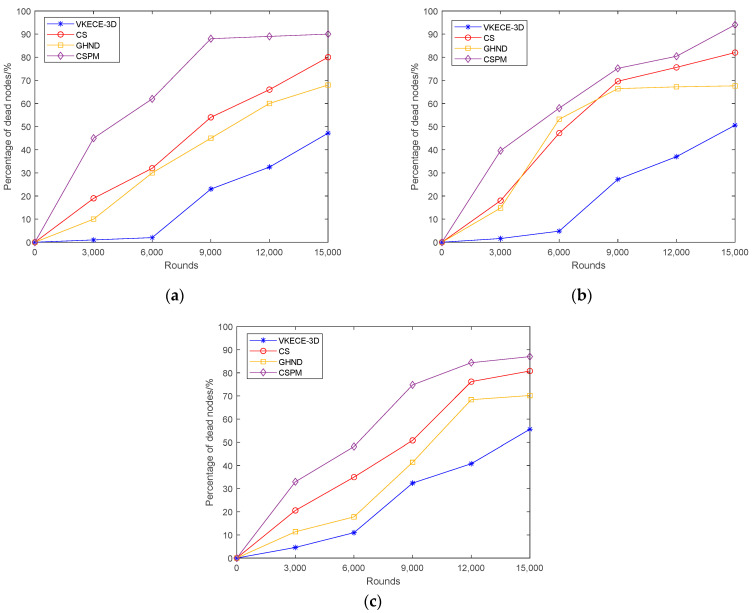
Node mortality of our proposed algorithm vs. comparison algorithms: (**a**) the small network, N = 100; (**b**) the medium-sized network, N = 250; (**c**) the large networks, N = 500.

To investigate the relationship between node mortality rate and the quantity of rounds of VKECE-3D, CS, GHND, and CSPM. The four algorithms are averaged after 100 independent experiments, and the experimental results for small, medium, and large networks are shown in Figure 15 were obtained. Analyzing Figure 15, we can see that the node mortality rate of all four algorithms grows with the quantity of iterations, but the node mortality rate of VKECE-3D has a significant advantage over the other three algorithms. Combined with Figure 11, Figure 12 and Figure 13 in Case 4, we can see that the node data multi-hop communication and polling working mechanism proposed in this research demonstrates how to successfully preserve node energy and extend the network life cycle.

#### 5.4.3. Activity Node Ratio

**Figure 16:**
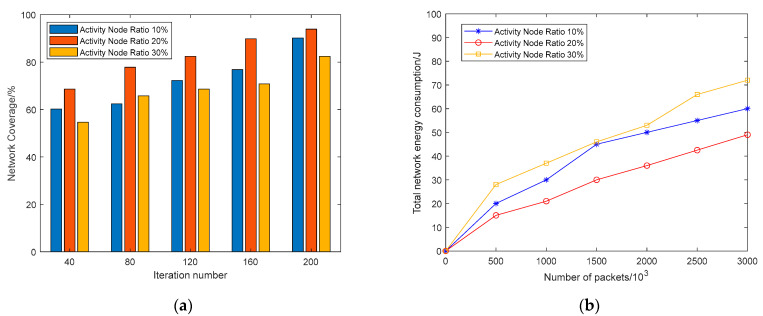
Determining the optimal ratio of activity nodes: (**a**) Coverage of different activity node ratios; (**b**) Total network energy consumption of different activity node ratios.

To determine the optimal percentage of active nodes, Figure 16 displays the results for coverage and total energy consumption for the whole network. Coverage of the network improves with more iterations, as shown in Figure 16a, and a network with 20% of active nodes achieves the best results for the same quantity of iterations. From Figure 16b, we observe that the total network energy consumption increases continuously with the quantity of packets sent, but the active node percentage at 20% consumes less energy compared to other cases. From the simulation results, the maximum coverage and lowest energy consumption may be attained by keeping the active node ratio around 20%, which is comparable to the value of the hyperparameter *k* that we selected in K-means, proving that the network has good performance when the elbow approach is used to choose *k* that has the same quantity of 3D-Voronoi cells.

## 6. Conclusions and Future Directions

With distributed base stations at the core and sensor nodes as IoT devices, the transmitted data aids in sensing coverage decisions via edge computing devices. One of the most pressing issues with HWSNs is how to enhance coverage in the area of interest. The coverage quality and life cycle determine the quality of service of HWSNs. The quality of coverage is mainly according to the network coverage. As a result, while deploying nodes, coverage quality is the most important consideration. We enhance the network coverage quality with the assistance of the highly destructive polynomial mutation strategy, the K-means algorithm, and the 3D-Voronoi partitioning strategy. The highly destructive polynomial mutation strategy is used for the re-deployment of nodes. The ideal sensing radius of nodes in 3D-Voronoi cells is computed using the K-means clustering algorithm and 3D-Voronoi partition strategy; the sensing radius of nodes is set by the distance between the center of mass and the cell boundary. This can achieve the full coverage of cells. While ensuring coverage, we also work on lengthening the network’s lifetime. Active nodes in the network may be kept to a minimum by defining the K-means clustering algorithm’s quantity of clusters as the quantity of 3D-Voronoi cells. The multi-hop communication and the polling working mechanism reduce the node’s energy consumption once the network is functioning properly, hence lengthening the network’s lifetime. Compared with existing 3D energy-efficient coverage enhancement methods, the VKECE-3D proposed in this research has better coverage and lengthens the network life cycle.

In this research, we mainly concentrate on the perceptual range heterogeneity of nodes and do not consider other node performance heterogeneity. Future research will optimize the HWSNs coverage by integrating multiple objectives into further considering complex real-world scenarios such as irregular areas, obstacle areas, and submarine mountains.

## Figures and Tables

**Table 1:** Notations and descriptions.

Notations	Descriptions
R	target area
$R_{s}$	sensing range
$R_{c}$	communication range
$R_{cov}$	coverage
k	quantity of K-means algorithm cluster
*SSE*	sum of the squared errors
Q	3D-Voronoi cell
BS	distributed base station

**Table 2:** Experimental parameters.

Parameter List	Value of Parameter
Size of the target area	50 × 50 × 50 $m^{3}$, 200 × 200 × 200 $m^{3}$, 600 × 600 × 600 $m^{3}$
Iteration number	250
Number of nodes deployed randomly	100, 250, 500
Initial energy of every sensor	5 J
Threshold battery power	0.02 J
Circuit unit energy consumption $\left(E_{elec}\right)$	50 nJ/bit
Free space channel parameter ($\varepsilon_{fs}$)	10 pJ/bit/m^3^
Multi-path channel parameter ($\varepsilon_{amp}$)	0.0013 pJ/(bit/m^4^)

## Data Availability

Not applicable.
